# Book Reviews

**Published:** 1907-05

**Authors:** 


### BOOK REVIEWS.

PHYSICAL DIAGNOSIS. With case examples of the Inductive Method. By Howard S. Anders, A. M., M. D., Professor Physical Diagnosis, Medico-Chirugical College, Philadelphia. Physician to the Philadelphia General Hospital, etc., etc. With eighty-eight illustrations in the text and thirty-two plates. D. Appleton & Co., New York.

Dr. Anders says in the preface that the average medical student tends to neglect or misapprehend the importance of two things, namely: technic and reason. He lacks method, patient procedure and practice, and his mental drift is toward jumping at conclusions from insufficient data and inefficient correlation. The purpose of his book is to counteract as far as possible this deficiency by insistence upon technical practice and precision and by inculcation of the inductice habit of thinking. Dr. Anders has attained considerable success in carrying out his plan and has written an excellent little book on Physincal Diagnosis.

It is divided into three parts. Part I takes up the chest and describes the various methods of elicitive information as to the condition of its organs; Part II describes the abdomen and its principle vicera, while Part III in on the Rontgen Ray in medical diagnosis. All the subjects are very satisfactorily covered in the 457 pages of text and index and makes of it a book that can be recommended to students and general practitioners of medicine.

---

A MANUAL OF OBSTETRICS. By A. F. A. King, M. D., Professor of Obstetrics and Diseases of Women in the Medical Department of the George Washington University, Washington, D. C., and in the Medical Department of the University of Vermont, etc. Tenth edition, enlarged and thoroughly revised. 12mo., 688 pages, with 30 Illustrations and three colored plates. Cloth, $2.75, net. Lea Brothers & Co., Philadelphia and New York, 1907.

King is one of the preennial books. It is now beginning its second quarter-century with its tenth edition and has thus spanned with vigor the most active and exacting period in medical history. No other obstetrical book extant has such a record. Every fact has a reason, and the ever-growing favor bestowed on King can have but one basis, namely merit. The author combines the faculties of a teacher and practitioner, and accordingly has been able to select what is important and to present it clearly. The student thus easily acquires a grasp of everything essential, and the accoucher can turn to these pages for reference on any point of practice. Suiting both classes of readers this single handy volume receives their combined demand and hence goes through successive editions, enabling the author always to keep it revised to date, as he has just done again, with considerable enlargement both in text and engravings, and with the addition of colored plates.

---

PROGRESSIVE MEDICINE, VOL. I, MARCH, 1907. A Quarterly Digest of Advance, Discoveries and Improvements in the Medical and Surgical Sciences. Edited by Hobart Amory Hare, M. D., Professor of Therapeutics and Materia Medica in the Jefferson Medical College of Philadelphia. Octavo, 280 pages, with illustrations. Per annum, in four cloth-bound volumes, $9.00; in paper binding, $6.00, carriage paid to any address. Lea Brothers & Co., Publishers, Philadelphia and New York.

The March issue of Progressive Medicine (Volume I, of the series for 1907) crystallizes the experience of a host of skilled observers, in all parts of the world, during the past year. In the opening pages Dr. Charles H. Frazier reviews the current literature of the Surgery of the Head, Neck and Thorax. The practical bearing of edema of the brain, in consideration of early operations for intracranial injuries, is carefully detailed.

Dr. Robert B. Predle deals with Infectious Diseases, including Acute Rheumatism and Croupous Pneumonia. The part taken by insects in the transmission of various infectious diseases is most carefully considerėd. Among other things, he says:

"If the Northern health officers were to make as vigorous a campaign against the house-fly, the bed-bug, and the flea, as the Southern officers have against the mosquito, the people would be freer from these diseases and, in addition, would be more comfortable."

Recent investigations prove that the tick is responsible for the transmission of spotted mountain fever and of relapsing fever. Under diptheria, over five hundred cases are reported by one observer in one year. The complications of the disease are deduced from an experience of over two thousand cases. Among these, pleurisy appears rarely. Dysentery, epidemic meningitis (from a study of 1,500 cases), malaria, measles (from a report of 1,205 cases), second attacks of pneumonia, the fresh-air treatment of pneumonia, rheumatism in childhood, scarlet fever, tuberculosis, typhoid fever, and yellow fever, all receive careful attention. Many important points are emphasized and new light thrown on obscure ones.

Dr. Floyd M. Crandall, in treating of the Diseases of Children, says:

"A troublesome obstacle that the pediatrist encounters to-day is the general ignorance and helplessnes of the young mother. She may be skilled in letters, art and sciences, as a college graduate, but may know little or nothing regarding the essential hygiene of early life."

Dr. D. Braden Kyle handles Rhinology and Laryngology with his customary skill and clearness.

Dr. B. Alexander Randall, in reviewing the literature of Otology for the past year, calls especial attention to the work which has been done on the labyrinth and its diseases. The study which Prof. Kubo, of Japan, has made on the relation of ocular movements to irritation of the labyrinth is most instructive. Labyrinth operations, meningitis, brain abscess (from a series of 645 cases), phlebothrombosis of the jugular vein and sinuses, transillumination of the mastoid, the blood-cloth dressing in mastoid operations and many other subjects are thoroughly presented.

